# Synthesis of HAp by Means of Sonoprecipitation Method

**DOI:** 10.3390/ma17133240

**Published:** 2024-07-02

**Authors:** Magdalena Stec, Piotr Maria Synowiec, Agnieszka Stolarczyk

**Affiliations:** 1 Department of Chemical Engineering and Process Design, Faculty of Chemistry, Silesian University of Technology, 44-100 Gliwice, Poland; piotr.synowiec@polsl.pl; 2 Inorganic Chemistry Research Group, New Chemical Syntheses Institute, Łukasiewicz Research Network, 44-100 Gliwice, Poland; 3 Department of Physical Chemistry and Technology of Polymers, Faculty of Chemistry, Silesian University of Technology, 44-100 Gliwice, Poland; agnieszka.stolarczyk@polsl.pl

**Keywords:** sonoprecipitation, hydroxyapatite (HAp), nanoparticles, ultrasounds (US), static mixer (STM)

## Abstract

Biomaterials, like hydroxyapatite (HAp), are the subject of many scientific investigations. Their specific application, however, is determined by the form and some characteristic features of the resulting material. Synthesis methods and optimization procedures leading to a product of predetermined characteristics are therefore of great interest. To broaden the existing knowledge, sonoprecipitation was investigated as a potential method for the production of nanosized HAp particles. The research was carried out in a static mixer (STM) immersed in the ultrasonic bath. The influence of operating conditions, e.g., ultrasonic power *P_US_* (*ε_US_*), ultrasonic frequency (f_US_), and unit mixing power (*ε_mix_*), was investigated in terms of nucleation intensity, product quality, and characteristics (particle size distribution (*PSD*), mean size, shape, etc.). As a result, the optimal conditions for the HAp nanoparticles synthesis (mean size: *d*~150 nm; length: *L*_1_~250 nm; width: *L*_2_~80 nm) in the form of needles/whiskers/rods—similar to the shape of the HAp present in natural human bones, free from agglomerates, with negligible signs of particle destruction—were determined. The formation of HAp of smaller sizes (*d* ≤ 100 nm) and more compact shapes (*L*_1_~155 nm, *L*_2_~90 nm), useful in bone regeneration processes, was also discussed.

## 1. Introduction

Hydroxyapatite (HAp) is a subject of interest to both scientists and manufacturers due to its outstanding properties like, e.g., bioactivity, biocompatibility, nontoxicity, osteoconductivity, etc., which account for its significant potential in a number of possible applications [1].

HAp is a chemical compound from the calcium phosphates group, which, from a thermodynamic point of view, is considered the most stable form (referring to physiological conditions such as temperature, pH, and the composition of body fluids) [2], and because of that, it is so eagerly used as a biomaterial, having direct contact with living organisms’ tissues. As to the most common applications of HAp, the literature [3,4] reveals the following:

- tissue engineering: HAp-coated implants are widely used in dentistry and orthopedics because HAp promotes the growth of a new bone towards the implant;
- drug/antigens/proteins delivery platforms, ensuring slow release rate and the protection of an active ingredient from degradation;
- bioimaging molecules/fluorescent dyes’ delivery: HAp is used for non-invasive imaging techniques of patients’ bodies [5,6], etc.

Hydroxyapatite may be synthesized through the use of various methods, including sol–gel, precipitation, dry methods, hydrothermal processes, etc. [4,7]. Each of them—showing both pros and cons—requires different procedures, different operating conditions, and different equipment to be used. From the above, crystallization with a chemical reaction (in other words chemical precipitation) is commonly used in practice due to the simplicity of the operation, relatively low costs, small amount of by-products, and the ability to control the particle size distribution of the product via the proper selection of process conditions [3,8,9].

The literature review shows that as starting solutions for the precipitation reaction, mostly aqueous solutions of (NH_4_)_2_HPO_4_ and Ca(NO_3_)_2_ [10,11], or, less frequently, of Ca(OH)_2_ and H_3_PO_4_ [11] or CaCl_2_ and Na_3_PO_4_ [12], are used. The analyzed sources are focused, among others, on the recognition of the influence of (i) solution pH, (ii) reaction temperature, or (iii) the use of additives modifying the characteristics of the final product [13,14,15]. These processes are carried out in both conventional stirred tank reactors [16] and Y-shaped reactors [10]. The use of static mixers with motionless mixing elements is not very popular; however, some reports are showing their application in practice. Particularly, the use of NETmix for HAp synthesis has been registered in the international patent WO2008/007992 A3 [17] and later discussed in the literature [18,19].

In the literature, one may also find information on the use of ultrasound-assisted precipitation for HAp production. In paper [20], the process is carried out in a specific chemical environment—a pseudo-body solution containing NaCl, KCl, NaH_2_PO_4_, KH_2_PO_4_, CaCl_2_, and MgCl_2_ used to increase HAp bioactivity. The US parameters are limited, to be specific, ultrasonic power *P_US_* is limited to one value equal to 100 W, and frequency to a range between 28 and 34 kHz. As a result, HAp nanoparticles of a spherical shape have been obtained. This, in turn, does not correspond to the experimental part of a proposed synthesis method and operating conditions. Another paper, the work of Hazar Yoruç and İpek [21], took into account the sonoprecipitation of HAp from different precursor reagents, showing that the size of particles decreases with the ultrasonic power increase up to 300 W. The cited work, however, did not consider the use of (NH_4_)_2_HPO_4_ and Ca(NO_3_)_2_ as reagents (as used in the presented paper).

From a practical point of view, it is mainly the methods that allow for the obtaining of hydroxyapatite in the form of nanoparticles that are of great interest, and they are still being sought. The reason for this lies within the general mechanical properties of ceramic materials which include HAp. It is known from the literature [22] that ceramics of sizes on the nanometer scale show better mechanical properties than the same substances with sizes on the microscale. The ability to produce nanosized HAp particles, however, requires the proper pH of the reaction environment. The literature indicates that for particles < 10 μm, the pH of the reaction should be maintained in the range of 8–9.3 [12]. Other authors report a pH value of ≥10 [10] or, more precisely, pH = 9.5–12 [4].

On the basis of the generally available data [23], one may find that HAp particles in the form of fibers, whiskers, or needles have better mechanical properties without affecting the biocompatibility of the obtained material; and what is more, such a shape is similar to HAp particles naturally present in human bones [24]; hence, the methods of synthesizing HAp with the above-mentioned shape are of general interest.

The product, HAp, should preferably take the form of single particles; that is, it should be free from agglomerated structures which, due to the presence of the mother liquid acting as a binder for individual grains, reduce the final purity of the product. Such an effect, i.e., a reduced number of agglomerates in the final product, may be achieved by the use of so-called modifiers, most of all in the form of dispersing agents. However, one should be aware of the fact that some of the modifiers/dispersants are toxic (such as sodium dodecyl sulfate (SDS) or hexadecyltrimethylammonium bromide (CTAB)) and cannot be used in applications where contact with living organisms is required. The others, like polyvinyl alcohol (PVA), despite the lack of contraindications in contact with living tissues, may significantly change the characteristics of reaction solutions (especially when their concentration is substantial, e.g., 5% *w*/*v* of PVA), and hence may significantly affect the nucleation kinetics (that were observed during the authors’ previous research carried out without the US support). Moreover, there is also a justified risk of a modifier presence in the final product (e.g., due to ineffective washing); consequently, a reduction in product quality could be observed. Another method that could be used to reduce/eliminate the agglomerates from the product—and a much less invasive one—is the use of ultrasound-assisted precipitation (so-called sonoprecipitation). Broad research carried out by the authors on the basis of CaF_2_ precipitation [25,26,27] has shown that the use of consciously selected ultrasound parameters may contribute to a significant reduction in the number of agglomerated structures, reduction of the mean particle size together with the *PSD* narrowing, and, depending on the precipitated chemical compound, changes in particles shape, which allows us to adjust the product features to actual requirements.

In light of the presented introduction, the main aims of the discussed paper are to (i) recognize the influence of ultrasounds (US) during the wet synthesis (precipitation) of HAp carried out in the in-line reactor with static elements (Koflo static mixer); (ii) expand the emerging database containing the results from the carried sonoprecipitation processes (based on different chemical compounds) to broaden the existing knowledge; and (iii) provide an optimal range of operating conditions (e.g., unit mixing power *ε_mix_* and US parameters—mainly power *ε_US_* and frequency *f_US_*) to obtain high-quality HAp in the form of nanosized particles with a narrow *PSD*, free from agglomerates and of whiskers/fibers/needle shapes.

One should also note that the presented research is only an introductory part of a deeper study and is focused only on the physical HAp features (e.g., mean size, particle size distribution (*PSD*), shape, presence of agglomeration phenomenon that could worsen the product quality, etc.) and its chemical composition. As authors, we are aware of the fact that the specific application (e.g., in one of the areas mentioned in the introduction) also depends on the product crystallinity, which may dedicate or exclude the synthesized HAp from further use, e.g., bone regeneration processes. Following that, we are now preparing a consecutive research stage that will include XRD (X-ray diffraction) analyses of the obtained HAp powders, as well as biocompatibility tests in a simulated body fluid (SBF). This step will answer the question of whether the ultrasounds have an impact on a particle structure and will help us to define possible applications of the developed production methods of HAp nanoparticles.

## 2. Calculations’ Basis

The methodology used for the calculation of turbulence intensity (based on the equivalent Reynolds number *Re_eqv_*) in reactors of different types, including static mixers (STMs), has been described in detail in [25]. The used definitions such as nucleation rates (*B*_0_) (in silent conditions (*B*_0*mix*_), in US-assisted conditions (*B*_0*US*_), as well as the relative value (*B*_0*rel*_)), relative input power (*ε_rel_*), and real residence time in STM (*t_m_*), together with the formulae used during calculations, may also be found in the authors’ previous paper [25].

## 3. Materials and Methods

For the synthesis of HAp, (NH_4_)_2_HPO_4_ (pure p.a., ACS, reagent Ph. Eur., POCH, Gliwice, Poland) and Ca(NO_3_)_2_·4H_2_O (extra pure, 98%, Thermo Scientific Chemicals, Fair Lawn, NJ, USA) have been used as starting solutions. To adjust the pH in the reaction system, 25% NH_3(aq)_ (pure p.a., POCH, Gliwice, Poland) has been used.

The experimental research took into account the sonoprecipitation of HAp carried out through the use of aqueous solutions of diammonium hydrogen phosphate (0.3 M) and calcium nitrate (0.5 M) (Equation (1)). The presented concentrations were selected on the basis of the literature review [10,14], as well as introductory research made in the silent system, and were a compromise between the mean particle size measured in nanoscale and the production yield.

As mentioned in the introduction, to ensure the synthesis of nanosized HAp particles, the strict pH of the reaction is one of the requirements to be fulfilled. Due to this, the pH of the diammonium hydrogen phosphate solution has been adjusted to the value of 9.5 through the use of 25% NH_3(aq)_ before the introduction of reagents into the reactor.

(1) $$6{\left(NH_{4}\right)}_{2}HPO_{4}+10Ca{\left(NO_{3}\right)}_{2}+2H_{2}O\to Ca_{10}{\left(PO_{4}\right)}_{6}{\left(OH\right)}_{2}+12NH_{4}NO_{3}+8HNO_{3}$$

The properties of the used starting solutions are presented in Table 1.

As the reaction system, Koflo static mixers (Koflo Corporation, Cary, IL, USA) with 6 semi-circular motionless inserts (length *L_m_ =* 0.186 m; internal diameter *d_m_* = 0.015 m), placed inside the ultrasonic bath (FALC Instruments, Treviglio, Italy, volume: 10 L; frequencies: 40/59 kHz; maximum power: 300 W; with the automatic adjustment in the range of 40–100% *P_max_*), were used. The selection of an in-line mixer with static elements was based on previous research, where the monodispersed product of a narrow *PSD* was obtained during the wet synthesis of CaF_2_ [25], which was also the goal of the presented HAp study. The US parameters like frequency *f_US_*, power *P_US,_*, and sonication time *τ_US_* were set on the ultrasonic bath control panel before the experiment. In all of the tests, the sonication time *τ_US_* equal to the mean residence time *t_m_* was used. Starting solutions, from the feed tanks, were introduced to the reactor simultaneously, with an equal volumetric flow rate, via the use of a pump (Masterflex driver with two Easy–Load heads, Avantor, Radnor, PA, USA, measuring range from 0.001 mL to 2900 mL). The measurements were carried out at room temperature (about 20 °C), controlled by the use of thermostats. The samples of the solid–liquid suspension, obtained as a result of the chemical reaction between the starting solutions, were collected when a steady state was reached. The separation of HAp particles from the mother liquid was performed via the use of a centrifuge (O’Haus, Nänicon, Switzerland, Frontier FC5816; max. speed: 15,000 rpm). The rotor speed was set to 4500 rpm and the operation lasted for 5 min. The filtrate was removed, and the solid was first washed with deionized water in the Soxhlet extractor and then dried at the temperature of 50 °C for at least 24 h.

**Figure 1:**
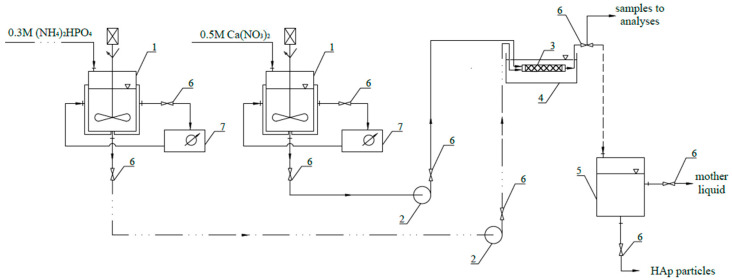
Scheme of the experimental setup: 1—tank for the starting solution (with a mechanical stirrer); 2—pump; 3—static mixer; 4—ultrasonic bath; 5—storage tank; 6—valve; 7—thermostat.

The scheme of the experimental setup is shown in Figure 1.

The parameters of the starting solutions (0.3 M (NH_4_)_2_HPO_4_ and 0.5 M Ca(NO_3_)_2_) and the filtrate (mother liquid) obtained after the solid separation from the solid–liquid suspension, e.g., density, dynamic viscosity, and pH, were determined by the use of standard laboratory equipment (densimeter DMA4500 Anton Paar, Graz, Austria, measuring range: 0–3 g/cm^3^; accuracy: ±5 × 10^−5^ g/cm^3^; viscometer ViscoLab400, Cambridge Viscosity, Inc., Boston, MA, USA, measuring range: (0.5–10) × 10^−3^; precision: ±1.5%; and pH meter Elmetron CP-401, Zabrze, Poland, precision class: ±0.002 pH).

The characteristics of the obtained HAp powder, namely, particle size, shape, and form (single particles/agglomerated structures), were determined via the use of the scanning electron microscope Phenom ProX (Thermo Fisher Scientific, Waltham, MA, USA) working with a back-scattered electron detector (BSD) (acceleration voltage 15 kV). The measurements of the single particle size were based on the analysis of SEM (scanning electron microscopy) images via the use of the freeware software ImageJ (version 1.53t), which is an accepted and commonly used processing method [28]. To be able to take micrographs of the obtained HAp nanoparticles that could be used for further digital analysis, the following sample preparation method was used: 1 mL of the collected suspension was introduced into the beaker and diluted 50 times with deionized water; next, the droplet of diluted suspension was placed directly on the aluminum sample table (to ensure good electrical conductivity of the substrate) and left to dry. Samples prepared in such a way were then inserted into the microscope, and the observations were made. The information about the size of single particles, as well as the *PSD*, was based on the measurements of 50–100 particles visible in the image/images. As the obtained particles had shown an elongated shape, two characteristic dimensions, namely, length *L*_1_ and width *L*_2_, were determined. Next, the mean size *d* was calculated as the equivalent diameter of the sphere of the same volume.

The purity and the chemical composition of HAp, based on the identification of functional groups present in the precipitated powders synthesized during experimental research, were determined with the Attenuated Total Reflection Fourier Transform Infrared Spectroscopy (ATR-FTIR). The ATR FT-IR spectra were recorded on a Spectrum Two spectrometer (PerkinElmer, Waltham, MA, USA) equipped with a deuterated triglycine sulfate *DTGS* detector. Spectra were recorded from an accumulation of 32 scans in the range of 4000–450 cm^−1^ with a resolution of 4 cm^−1^ with a clean ATR diamond crystal used as a reference.

The experimental research took into account the variability of the unit mixing power (*ε_mix_*), ultrasonic frequency (*f_US_*), and ultrasonic power (*P_US_*) recalculated to the form of the unit power (*ε_US_*). The range of the considered operating conditions is presented in Table 2.

To ensure the results’ repeatability, each of the syntheses has been carried out 3 times.

## 4. Results and Discussion

As mentioned previously, this paper aims to recognize the influence of mixing intensity together with the influence of ultrasounds’ action on the synthesized HAp particles (e.g., mean particle size, *PSD*, shape, product purity, presence of the agglomerated structures, etc.) and to define the optimal working conditions leading to the formation of a monodispersed product in the form of single nanoparticles, free from agglomerated structures (or at least with their reduced number). Because the used ultrasonic device works in the low-frequency region dedicated to the nucleation step intensification [29], the analysis of the nucleation rate is also presented.

### 4.1. The Role of Ultrasonic Power ε_US_

To enable the recognition of the ultrasonic power influence on particle characteristics, all of the measurements were carried out under constant unit mixing power (*ε_mix_ =* 0.1 W/kg). Initially, HAp sonoprecipitation experiments were carried out with four testing values of the ultrasonic input power (*ε_US_* = 11.62, 14.52, 21.78, 29.04 W/kg → experiments 1–4 (Table 2)) to find the optimum, on the one hand enabling the reduction of agglomerates number in the population, and on the other preventing single particles from the excessive fragmentation. On the basis of the digital measurements of crystal sizes (*L*_1_ and *L*_2_) in the collected SEM micrographs, carried out in ImageJ software, the *PSDs* were determined via the use of OriginPro (version 8.0), and the mean particle size *d* has been calculated for each synthesis method considered. The determined values of *d* are next presented as a function of *ε_US_* on the scatter chart and fitted by the use of a 3rd-degree polynomial function. It was observed that the smallest primary particles can be obtained in the unit power range between 14.52 and 21.78 W/kg. Through the use of the minimal particle size criterion, the new value of the ultrasonic power has been estimated. It was found that a value of approximately 16.65 W/kg (which corresponds to *P_US_* = 172 W) fits the criterion. To facilitate the programming of the ultrasonic bath, the unit power input *ε_US_* = 16.46 W/kg (*P_US_* = 170 W) was added as another parameter to be tested (experiment 5, Table 2).

**Figure 2:**
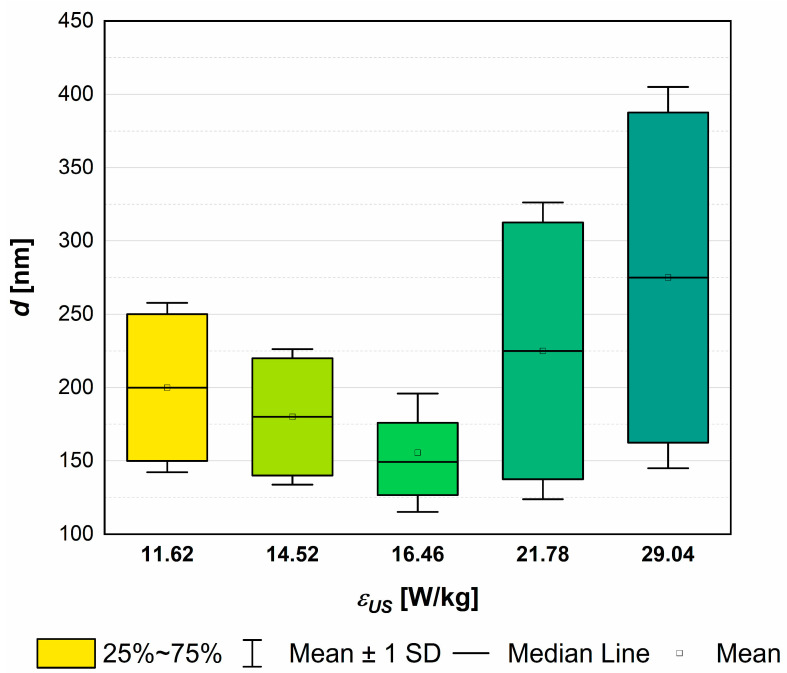
The influence of the unit ultrasonic power *ε_US_* on the *PSD* and the mean particle size *d*. Operating conditions: *f_US_* = 40 kHz; *ε_US_* = 0.1 W/kg.

**Figure 3:**
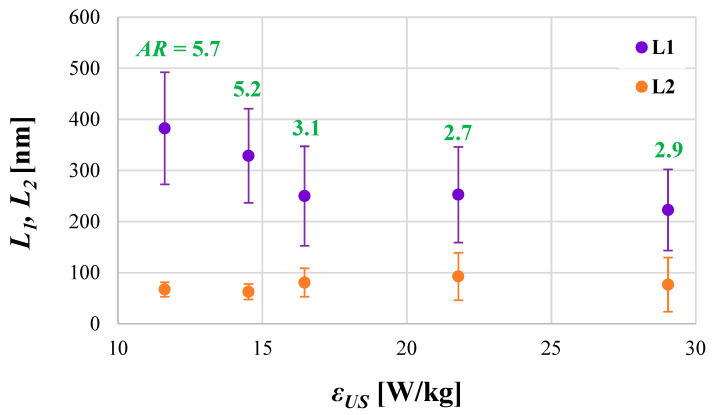
The influence of the unit ultrasonic power *ε_US_* on the particles’ length *L*_1_ and width *L*_2_ together with corresponding aspect ratio *AR* values. Operating conditions: *f_US_* = 40 kHz; *ε_mix_* = 0.1 W/kg.

In Figure 2 and Figure 3, the characteristic parameters used to define the size (e.g., length (*L*_1_), width (*L*_2_), mean particle size (*d*)) and general shape (aspect ratio (*AR*) = *L*_1_*/L*_2_) of the obtained HAp particles as a function of the unit ultrasonic power (*ε_US_*) are shown.

From the presented Figure 2, one may observe that there is an optimum of the ultrasonic power which allows for the obtaining of single particles with a reduced size and a relatively narrow *PSD* that are not subjected to excessive destruction (which, in turn, would lead to secondary sonoagglomeration, contaminating the final product with the mother liquid). The present findings confirm the conclusions reached previously during studies on the sonoprecipitation of CaF_2_ [25,26,27], making them more general and very useful in practice.

**Figure 4:**
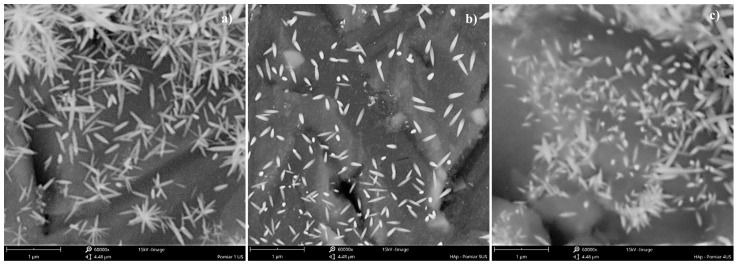
SEM images of HAp particles precipitated in the Koflo static mixer. (**a**) *ε_US_* = 11.62 W/kg; (**b**) *ε_US_* = 16.46 W/kg; (**c**) *ε_US_* = 29.04 W/kg. Operating conditions: *ε_mix_* = 0.1 W/kg; *f_US_* = 40 kHz. Size bar: 1 μm (magnification: 60,000×).

As shown, the use of a US unit power of 16.46 W/kg (in combination with other parameters such as *f_US_* = 40 kHz and *ε_mix_* = 0.1 W/kg) allows us to limit the mean particle size to approx. 150 nm and to obtain the population of particles with the narrowest *PSD*. A further increase in the ultrasonic power (values 21.78 W/kg and then 29.04 W/kg) results in a harsh destruction of primary particles. It is no longer the case that the agglomerated structures are broken (compare to the power values of 11.62 W/kg and 14.52 W/kg, which, in fact, were insufficient to break all of the clusters (see SEM images in Figure 4)), but it looks like the primary particles are fragmented. The expansion of the *PSD* distribution is a result of both the fragmentation of the primary particles and the secondary sonoagglomeration, which merges fragments with each other or with other primary particles. This is why an increase in the mean particle size *d* is observed.

Taking into account the particles’ shape, one may base the observations connected with HAp particles on the insights made during previous sonoprecipitation studies, namely, that such a feature as a shape is affected by both mixing conditions (*ε_mix_*) and the ultrasonic power (*ε_US_*). When the system works in silent conditions (there is no US support), shape is determined via the turbulence intensity caused by mixing. For mild mixing conditions, in which particles are not subjected to destruction caused by particle–particle or particle–static inserts’ collisions leading to breakage, the shape remains unchanged. However, when turbulence intensity increases (and that may be caused by (i) the increase in the unit mixing power; (ii) the introduction of US to the reaction system where the implosion of cavitation bubbles is responsible for the formation of additional structures (e.g., swirls, vortices, etc.) of turbulent flow; or (iii) a combination of the above), the initial shape may be altered. As mentioned, the HAp experiments including the change in ultrasonic power (*ε_US_* = vary) were carried out for the constant value of the unit mixing power (*ε_mix_ =* 0.1 W/kg). For such a value, however, when a system had remained in silent conditions (i.e., there was no US support), HAp particles showed an elongated shape (needles/whiskers/rods). The same shape was observed when experiments with different values of ultrasonic power were carried out though different aspect ratios (*AR*) (defined as *L*_1_*/L*_2_*)* were noted. As shown in Figure 3 and Figure 4, low values of ultrasonic power lead to the formation of long, thin particles (*AR* about 6–5 for *ε_US_* ≤ 14.52 W/kg) in the shape of whiskers/needles with sharp ends that tend to form bundles of needles/stars/chrysanthemum structures and are often agglomerated in clusters (Figure 4a). For the optimal *ε_US_* = 16.46 W/kg (Figure 4b), single particles in the shape of rice grains, visibly shorter, with *AR* reduced to approx. 3.4 (the length *L*_1_ is significantly reduced at the small expense of the width *L*_2_ (Figure 3)), were obtained. It is also very important that a complete reduction of agglomerated clusters was achieved. Nevertheless, some subtle signs of particles’ destruction may be also observed. Primary particles undergo some slight damage, which causes a decrease in the mean particle size *d* (Figure 2). Due to this, one may see smaller particle fragments (some even of a shape close to the sphere), which is a result of single particles’ fragmentation (breakage) and the abrasion of the edges (Figure 4b). When *ε_US_* is greater than the optimal value (e.g., 29.04 W/kg (Figure 4c)), a negative impact of the increased turbulence on both *PSD* and particles’ shape is visible. The population is non-uniform; one may notice elongated particles, together with those of a rice shape, as well as small, almost spherical ones. The average *AR* is at the level of 3; however, the relative error is in the range of 1.68–88.94% (the average value is at the level of 32.5%). The secondary sonoagglomeration is also present.

The described observations will also be confirmed during the further recognition of the changes in the nucleation rate caused by the change of operating conditions, namely, *ε_US_.*

The presented analysis confirms that the ultrasonic power *ε_US_* is a key parameter influencing the characteristics of the crystalline product. One should be aware that the conscious selection of *ε_US_* is a milestone in enabling the production of particles with desired features.

### 4.2. The Role of Ultrasonic Frequency f_US_

The role of the ultrasonic frequency in a reaction system depends on both the used range of *f_US_*, determining the dominant stage in the crystallization process (nucleation or particle growth), and the resonant size of cavitation bubbles, influencing the strength of implosion.

In the literature [29,30,31,32], one may find information about the so-called low- (20–100 kHz) and high-frequency (up to 5 MHz) regions. A low-frequency region is used when there is a need to intensify the nucleation step, and in turn, a high-frequency region is responsible for promoting particle growth. As the precipitation of HAp is an example of a fast ionic reaction directed to the formation of nanoparticles, the low ultrasonic frequencies (40 and 59 kHz, available in the selected ultrasonic bath) were selected as testing values. The corresponding sizes (radii) of cavitation bubbles generated in the system, namely, 75 and ~51 μm, were calculated on the basis of Minaret’s equation [33].

**Figure 5:**
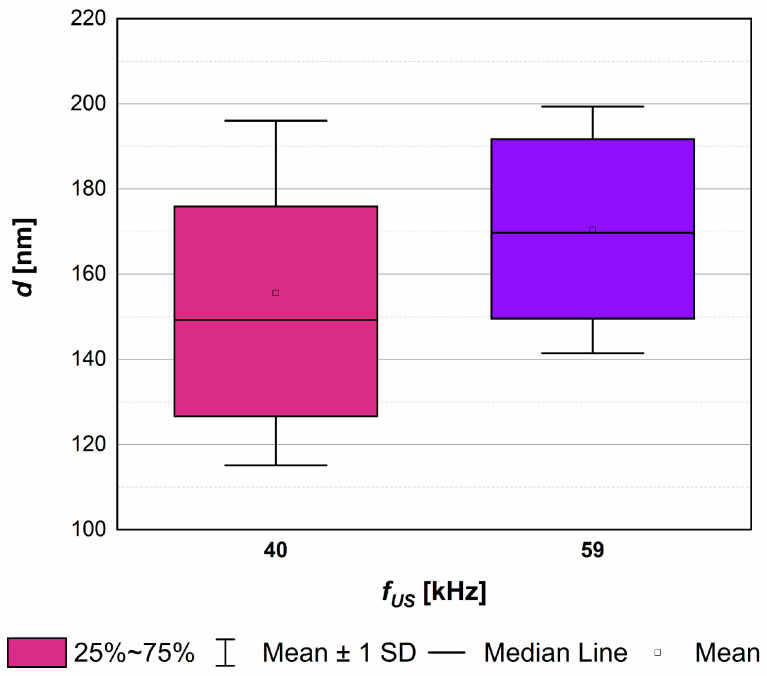
The influence of ultrasonic frequency *f_US_* on *PSD* and the mean particle size *d*. Operating conditions: *ε_mix_* = 0.1 W/kg; *ε_US_* = 16.46 W/kg.

**Figure 6:**
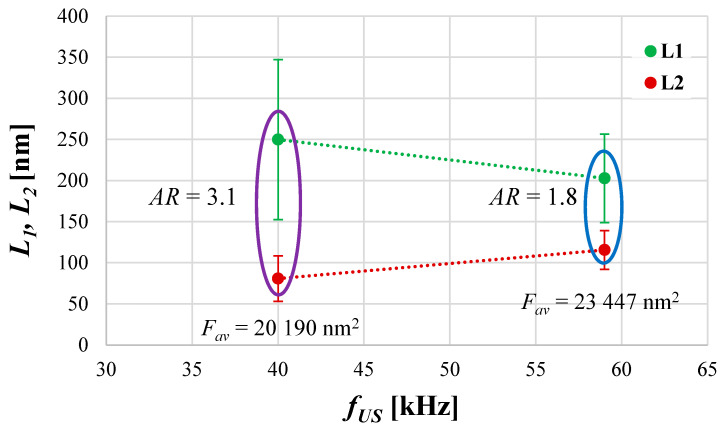
The influence of ultrasonic frequency *f_US_* on the particles’ length *L*_1_ and width *L*_2_ together with values of the average area of particles’ surface *F_av_*. Operating conditions: *ε_mix_* = 0.1 W/kg; *ε_US_* = 16.46 W/kg.

The experimental research, taking into account the influence of *f_US_* on HAp precipitation results, was carried out for a constant unit mixing power (*ε_mix_ =* 0.1 W/kg), and the selected optimal *ε_US_* = 16.46 W/kg. Figure 5 and Figure 6 present the obtained results, showing the mean particle size *d*, as well as characteristic dimensions of HAp particles *L*_1_ and *L*_2_ (together with *AR*) as a function of ultrasonic frequency *f_US_*.

On the basis of the presented results (Figure 5), one may notice that as the ultrasound frequency increases, the mean size of HAp particles increases slightly. The explanation for such behavior lies within the size of cavitation bubbles. As mentioned at the beginning of this section, the tested ultrasonic frequencies, 40 and 59 kHz, led to the formation of cavities with a size of about 75 μm and 51 μm, respectively. This means that lower frequencies are responsible for the generation of bigger bubbles. The greater the bubbles are, the more violent is their implosion, and the stronger the destruction of particles caused by a significant increase in turbulence intensity observed. This results in the formation of particles with a reduced mean size *d*, together with multiple fragments, which, in turn, broadens the *PSD*. Even if, at first glance, Figure 6 does not seem to be consistent with the data presented in Figure 5, the comparison of the average particles’ surface area *F_av_* (used for the calculation of a mean particle size) confirms the presented data convergence. What is more, similar conclusions may be found in the literature connected with the sonocrystallization processes carried out by other scientists [29,30,34], as well as in the papers published previously by the authors considering the sonoprecipitation of CaF_2_ [25,26,27]. It should also be mentioned that due to a small difference between the analyzed *f_US_*, the observed reduction in the mean particle size (from 170 nm to 158 nm) was also limited (approx. 6%).

**Figure 7:**
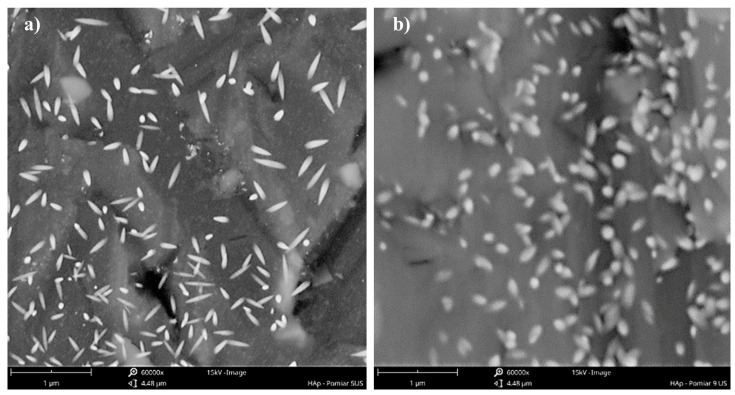
SEM images of HAp particles precipitated in the Koflo static mixer: (**a**) *f_US_* = 40 kHz; (**b**) *f_US_* = 59 kHz. Operating conditions: *ε_mix_* = 0.1 W/kg; *ε_US_* = 16.46 W/kg. Size bar: 1 μm (magnification: 60,000×).

**Figure 8:**
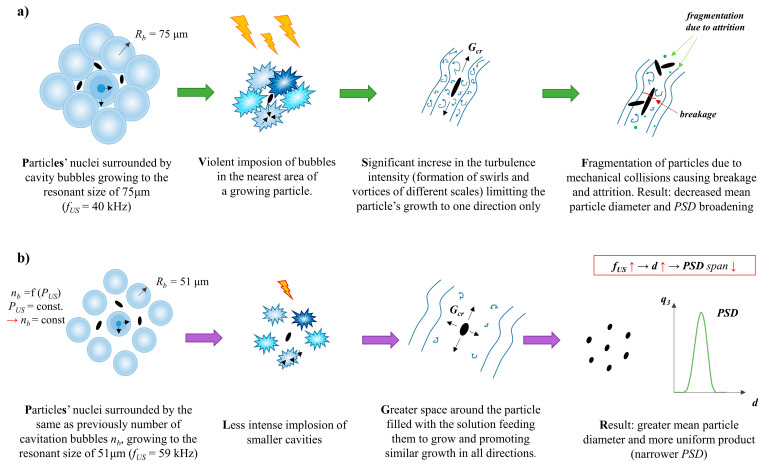
The graphical representation of a possible mechanism causing the change of particles’ shape with the change of the ultrasonic frequency: (**a**) *f_US_* = 40 kHz; (**b**) *f_US_* = 59 kHz.

It is also very interesting that a simultaneous decrease in both characteristic particle sizes, length *L*_1_, and width *L*_2_ (Figure 6) is observed when the ultrasonic frequency increases to 59 kHz. Accordingly, some differences in the particles’ shape and *AR* are noticeable (Figure 7). In the analyzed cases (experiments 5 and 6, Table 2), the turbulence intensity, described by the equivalent Reynolds number, was at the same level (*Re_eqv_* = 6483) as the constant values of the unit mixing power *ε_mix_* and ultrasonic power (*ε_US_*), the main parameters influencing the shape, were selected. For such operating conditions, where the only variable is the ultrasonic frequency, it would seem that the shape of particles should be preserved. Nevertheless, the change in *f_US_* caused the formation of HAp particles of a slightly differentiated morphology. For *f_US_* = 40 kHz, the obtained particles resemble long-grain rice (an oblong shape with sharpened ends (Figure 7a)) with a greater *L*_1_/*L*_2_ ratio (*AR*~3.1), while for *f_US_* = 59 kHz, the particles’ shape is close to oval (with more rounded ends (Figure 7b)), and there is no longer such a significant change in their dimensions *L*_1_ and *L*_2_ (*AR*~1.8). As, for now, the literature does not describe such findings, and no explanation has yet been found in the relevant data; we may only suggest that in the case of a lower ultrasonic frequency used (*f_US_* = 40 kHz), there were a great number of cavities (the same in both considered cases as the ultrasonic power, which affects the number of generated bubbles, was constant) of bigger size, and due to this, they implode more violently, causing a reduction in the space where the nuclei grow until they reach a critical size. This is why growth is promoted mainly in one direction, corresponding to the particle length. On the other hand, when a greater ultrasonic frequency is used (*f_US_* = 59 kHz), smaller cavitation bubbles are generated. Their implosion is less violent, and the induced turbulence structures are less intense. The free space, filled with a solution feeding the nuclei to grow, is reduced to a smaller extent, and the formation of particles with comparable sizes *L*_1_ and *L*_2_ is favored. The graphical representation of the proposed idea is shown in Figure 8a,b. We have also considered the scenario in which the particle stays inside the growing bubble; however, in our opinion, it is rather unlikely, primarily due to the chemical composition of Hap, which contains hydroxy ions. These make the HAp a hydrophilic compound that is readily wetted by the aqueous solution. However, the cavitation bubble is filled with a gas (vapor), and this is why the energetic state of HAp would be unfavored. What is more, from a thermodynamic point of view, the energy associated with the formation of a cavitation bubble is proportional to the phase boundary surface area. Hence, the bubbles should form preferentially on the particles’ surface. However, given the small particle size compared to that of bubbles, we expect this effect to be rather negligible. The latest scientific report based on flotation [35] shows a strong relation between the particles’ shape and the gas bubble formation. The performed bubble–particle attachment tests carried out by the team of Guangxi Ma showed that the attachment efficiency is in the order of cubic > triangular > cylindrical > spherical. What is more, sharp particle edges also result in increased attachment. Assuming that it is possible to transfer the results directly to the cavitation process, such findings may make us believe that for lower ultrasonic frequencies, particles are more tightly surrounded by cavities; thus, the presented idea (shown in Figure 8) could be correct. Accordingly, there might be a relation between the shape of the particles and the ultrasonic frequency; however, the presented explanation is just a hypothesis, which requires further recognition, and this is why we are open for discussion.

### 4.3. The Role of Unit Mixing Power ε_mix_

As described in detail in [36,37], there is a strong link between the fluid-dynamic conditions and characteristics of the obtained solid product. The properly selected mixing conditions, together with US parameters (when the system is supported by ultrasonic waves) or without them (when silent conditions are considered), affect the turbulence intensity, which, on one hand, may help to accomplish the following:

- reduce the agglomeration phenomenon (if the destruction stress, resulting from mixing on static elements or a combination of mixing and the additional use of US, exceeds the agglomerates’ tensile strength);
- lower the mean particle size;
- change the shape of the particles (which can either be considered an advantage or a disadvantage);
- alter the nucleation kinetics,

but on the other hand, they may lead to the following:

- excessive particle destruction (due to intense breakage);
- reduction of product purity due to the secondary sonoagglomeration phenomenon.

This is why a proper combination of the above-mentioned mixing conditions and US parameters is of great importance during the sonocrystallization/sonoprecipitation processes.

In the considered research, all of the experiments aimed at the recognition of the mixing conditions on HAp particles’ characteristics were carried out for the previously selected *ε_US_* = 16.46 W/kg and *f_US_* = 40 kHz, which enabled obtaining a HAp particle population with a narrow distribution, free from agglomerates and of needle/whisker shapes.

**Figure 9:**
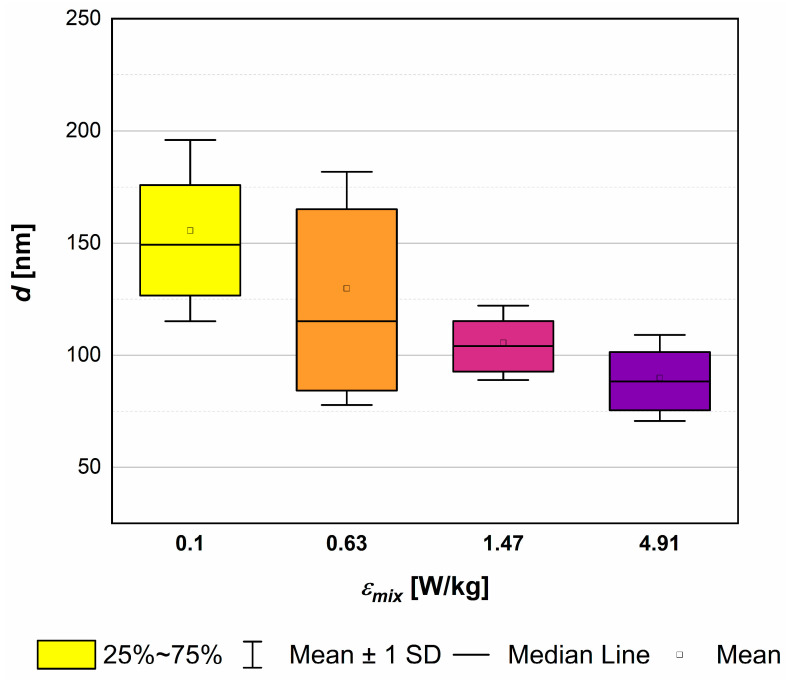
The influence of unit mixing power *ε_mix_* on the *PSD* and the mean particle size *d*. The corresponding values of *Re_eq_*_v_ (from the left): 6483, 6552, 6657, 7058 (see Table 2). Operating conditions: *f_US_* = 40 kHz; *ε_US_* = 16.46 W/kg.

**Figure 10:**
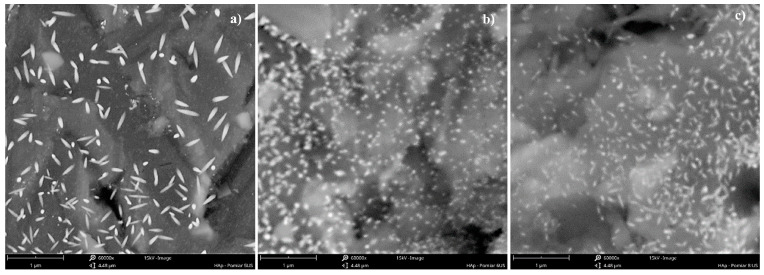
SEM images of HAp particles precipitated in the Koflo static mixer: (**a**) *ε_mix_* = 0.1 W/kg; (**b**) *ε_mix_* = 1.47 W/kg; (**c**) *ε_mix_* = 4.91 W/kg. Operating conditions: *f_US_* = 40 kHz; *ε_US_* = 16.46 W/kg. Size bar: 1 μm (magnification: 60,000×).

**Figure 11:**
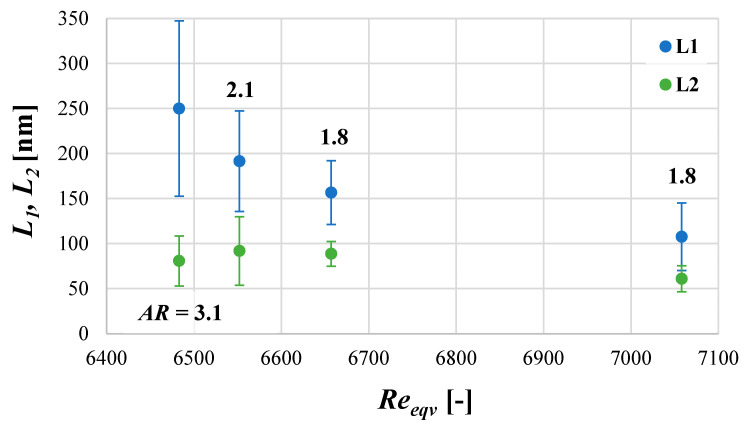
The influence of mixing conditions (represented by *Re_eqv_*) on the particles’ length *L*_1_ and width *L*_2_ together with particles’ population aspect ratio *AR*. Operating conditions: *f_US_* = 40 kHz; *ε_US_* = 16.46 W/kg.

The results obtained via the use of four different values of the unit mixing power—*ε_mix_* = (0.10, 0.63, 1.47, 4.91) W/kg—are presented in Figure 9, Figure 10 and Figure 11. The corresponding values of the equivalent Reynolds number *Re_eqv_* may be found in Table 2.

As shown in Figure 9, the increase in mixing intensity leads to a significant reduction in a mean particle size *d* (from the initial 150 nm for *ε_mix_* = 0.1 W/kg up to ~90 nm for *ε_mix_* = 4.91 W/kg). What is also clear is that changes in turbulence intensity, induced by the change in *ε_mix_* at a constant *ε_US_*, may affect the span of particle distributions. From the examined conditions, taking as a criterion the narrowest *PSD* and relatively low size *d*, the most favorable one seems to be obtained when *ε_mix_* = 1.47 W/kg (*d*~105 nm). However, in practice, slight problems with STM clogging had started and periodic washing was required. Some changes in particles’ shape, from quite long needles/whiskers (Figure 10a) to more oval ones (Figure 10b) with the *L*_1_ and *L*_2_ dimensions closer to each other (*AR*~1.8) (Figure 11), have been observed. Further, the increase in *ε_mix_* up to 4.91 W/kg, on the one hand, caused a further reduction in the mean particle size (*d*~90 nm); but on the other, a slight simultaneous expansion of the *PSD* was observed. This was caused by particles’ destruction and the fragmentation of primary particles into smaller pieces, visible in Figure 10c. Although it is not so clear from the image, these conditions (*ε_mix_* = 4.91 W/kg; *ε_US_* = 16.46 W/kg; and *f_US_* = 40 kHz) were the beginning of the secondary sonoagglomeration phenomenon. It was discovered during initial experiments after FT-IR analyses because the product was contaminated by ammonium nitrate NH_4_NO_3_ and required more thorough washing. Moreover, much more intense clogging was also observed. This highlighted the necessity of the reactor’s washing after several (2–3) measurement series. In the case of continuous processes, this may lead to decreased productivity as auxiliary operations would then be required. Regarding the particles’ shape, it is similar to previous conditions; however, the length *L*_1_ is visibly smaller at the expense of the width *L*_2_, which results in a similar aspect ratio (*AR*~1.8) (Figure 11).

### 4.4. The Role of Operating Conditions on Nucleation Intensity B_0_

**Figure 12:**
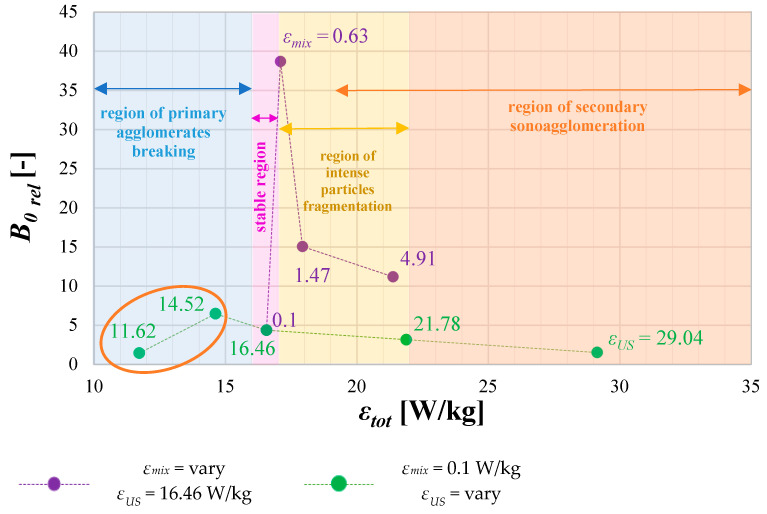
Relative nucleation rate *B*_0*rel*_ as a function of total input power *ε_tot_* (*f_US_* = 40 kHz).

Taking into account the possible change in the nucleation rate with the change in turbulence intensity induced either by the change in ultrasonic power *ε_US_* or the mixing conditions *ε_mix_*, Figure 12 is presented, showing the relation between the relative nucleation rate *B*_0*rel*_ and the total input power *ε_tot_*. On the basis of the given data, one may suggest that the role of the mixing input power *ε_mix_* (combined with the optimal value of the ultrasonic power) in nucleation rate intensification is even greater than the role of ultrasonic power itself, which is strictly connected with the characteristic, elongated, needle-like shape of the obtained HAp particles. In the considered conditions, when different values of *ε_US_* were examined (*ε_mix_* = 0.1 W/kg), the maximum increase in the nucleation rate was at the level of 6.5 (green series) and was reached as a result of primary agglomerates destruction (orange oval in Figure 12); while for different values of *ε_mix_* (and constant value of *ε_US_* = 16.46 W/kg), the increase reached 40 (violet series), and that, in turn, was an effect of intense particle fragmentation due to mechanical collisions. The presented information confirms that the reciprocal relation between the ultrasonic power (selected in the first step as a crucial parameter) and mixing conditions, represented as the total input power *ε_tot_*, may significantly influence the nucleation stage of the process. The collected data (Figure 12) allowed for the designation of four characteristic areas of *ε_tot_*, showing some specific changes in the relative nucleation rate caused by different mechanisms. When *ε_tot_* ≤ 16 W/kg, the increase in *B*_0*rel*_ is caused mainly by the intense destruction of primary agglomerates down to single particles obtained at the end of the mentioned section. The region where *ε_tot_* stays at the level between 16 and 17 W/kg (16 < *ε_tot_* ≤ 17) corresponds to a stable region, without any negative impact on HAp particles due to phenomena like destruction or secondary agglomeration. A slight increase in the relative nucleation rate observed (*B*_0*rel*_~5) is just a result of US introduction to the reaction system (in comparison to silent conditions) of properly selected parameters (agglomerated clusters break into single primary particles, without any signs of fragmentation). Further increase in *ε_tot_* (*ε_tot_* > 17 W/kg) results in a decline of *B*_0*rel*_, caused by intense single particle fragmentation and then by the secondary sonoagglomeration phenomenon in which particles’ fragments merge together with the use of the mother liquid acting as a binder. One should be aware of the fact that the presented limits of *ε_tot_*, dividing the area between fragmentation and secondary agglomeration, are not hard-coded and may change since these phenomena may overlap (which is symbolically indicated by the extension of the arrow of the secondary agglomeration region). What should be also explained is that the phenomenon of breaking single particles into smaller fragments is much more serious with the change of mixing conditions than the change in ultrasonic power *ε_US_*. One may explain such behavior using the characteristic needle shape of the precipitated HAp particles and their nanometer-scale size; that is, during the flow of liquid–solid suspension through the reactor with motionless inserts, elongated particles easily undergo mechanical destruction due to collisions (with walls, with static elements, and with each other). At the beginning, the destruction is very harsh (what is observed when *ε_mix_* is increased from 0.1 to 0.63 W/kg), particles break into smaller ones which, in turn, are less and less susceptible to destruction, and this is why a decline in *B*_0*rel*_ is observed—first from *ε_mix_* = 0.63 to 1.47 W/kg, which is clearer, and then from *ε_mix_* = 1.47 to 4.91 W/kg, which is less significant. If it is about the influence of ultrasonic power, it is much less important in the considered case, and the main cause lies within the size of both the cavity bubbles and the HAp particles. The radius of cavity bubbles, for the considered frequency, is measured in micrometers, when HAp particles are measured in nanometers. This means that the linear size of the bubbles may be up to 1000 times greater (when, again, we would compare their volume, the difference increases to the value of the order of 10^9^), and due to this, their implosion is less harmful to particles than their direct contact with obstacles.

As shown (Figure 12), for the considered range of operating conditions, to cause a significant increase in the nucleation rate of HAp particles (in comparison to the system working without US support), *ε_tot_* should be kept at the level of about 17 (in the considered reaction system, it relates to *ε_mix_* = 0.63 W/kg and *ε_US_* = 16.46 W/kg), which corresponds to the intense primary particles fragmentation region. Such an observation is consistent with previous data (Figure 9), where for *ε_mix_* = 0.63 W/kg, the *PSD* was the broadest due to breakage and fragmentation of single particles. A further increase in *ε_mix_* no longer resulted in such significant changes in *B*_0*rel*_ as already broken, initially elongated particles acquired a more compact shape (*L*_1_ comparable to *L*_2_), which is much more difficult to destroy.

**Figure 13:**
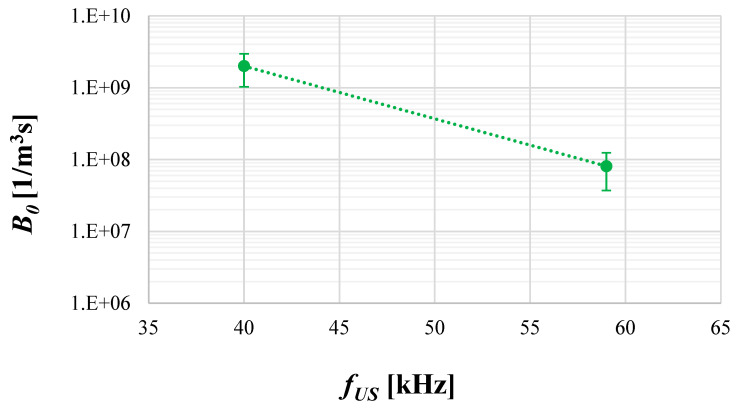
Nucleation rate *B*_0_ as the function of ultrasonic frequency *f_US_*. Operating conditions: *ε_mix_* = 0.1 W/kg; *ε_US_* = 16.46 W/kg.

Now, moving to the influence of ultrasonic frequency on the nucleation rate (Figure 13), one may observe that when the US frequency increases, a decrease in nucleation intensity (from approx. 2 × 10^9^ to 1 × 10^7^) is noted. The explanation is connected with the intensity of cavitation bubbles’ implosion which decreases when the used *f_US_* increases. This, in turn, results in reduced particles’ destruction; hence, a lower intensity of secondary nucleation generated by fragments of primary particles is observed, which affects the final value of *B*_0_. Such observations are in agreement with other literature reports (e.g., [38]).

**Figure 14:**
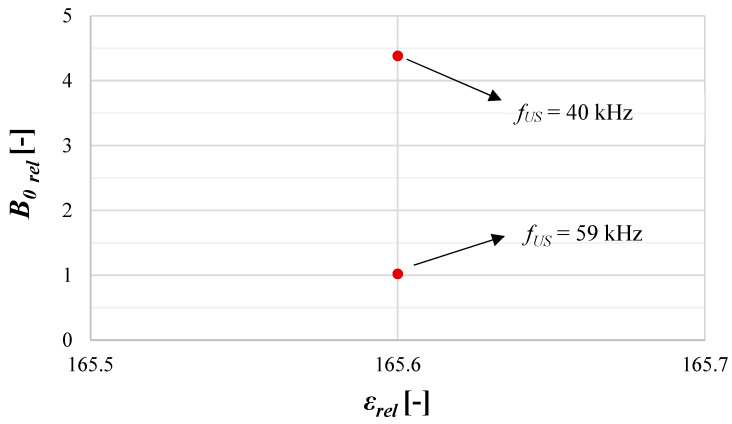
The intensification of the relative nucleation rate *B*_0*rel*_ by the use of a lower ultrasonic frequency *f_US_*. Operating conditions: *ε_mix_* = 0.1 W/kg; *ε_US_* = 16.46 W/kg.

Summing up, the use of lower US frequencies (40 kHz in the considered research) may help us to intensify the nucleation stage of the precipitation process. It is visible in Figure 14, in which the relative nucleation rate *B*_0*rel*_ is presented for the same mixing conditions (*ε_mix_* = 0.1 W/kg), and a constant US power (*ε_US_* = 16.46 W/kg) for both of the tested ultrasonic frequency values. It is clear that the reduction of *f_US_* to 40 kHz allowed for an increase in nucleation rate of about 4.5 times compared to the case when *f_US_* = 59 kHz was used.

### 4.5. The Mean Product Size d as a Function of Turbulence Intensity

**Figure 15:**
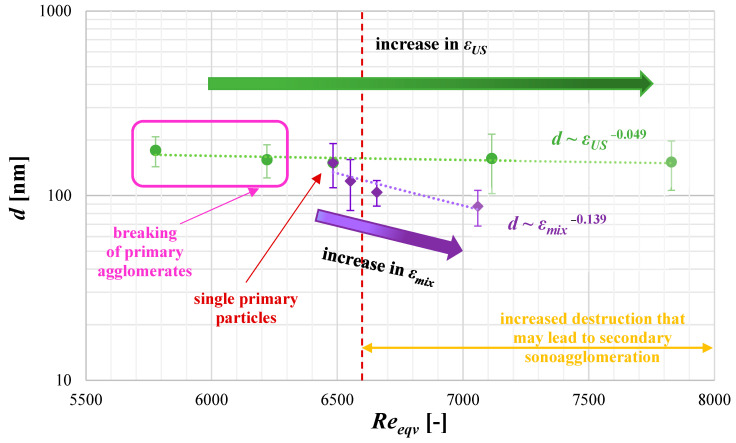
The mean particle size *d* as a function of the relative input power *ε_rel_* (*f_US_* = 40 kHz).

To sum up the considerations concerning the influence of turbulence intensity induced by mixing and US assistance (*Re_eqv_*) on the mean particle size *d*, Figure 15 is presented.

The given data (Figure 15) illustrate once again the crucial importance of operating conditions selection. As shown, only the proper mutual relation between the ultrasonic power and mixing conditions (*Re_eqv_* = 6300–6500) allows of the obtaining of single, primary particles, free from agglomerated clusters, without noticeable signs of particles’ destruction. What is also clearly visible, and which stays in agreement with previous conclusions made on the basis of nucleation kinetics recognition, is that in the case of elongated particles’ destruction, leading to fragmentation, is caused mainly by mechanical collisions due to increased unit mixing power. As in the considered case, the difference between the size of the cavity bubbles and the particles is in the order of 10^3^ (in favor of cavities); the influence of ultrasonic power in breakage is less significant, and this is why only negligible changes in the mean particle size are observed.

### 4.6. Powder Characterization—Chemical Composition and Product Purity

**Figure 16:**
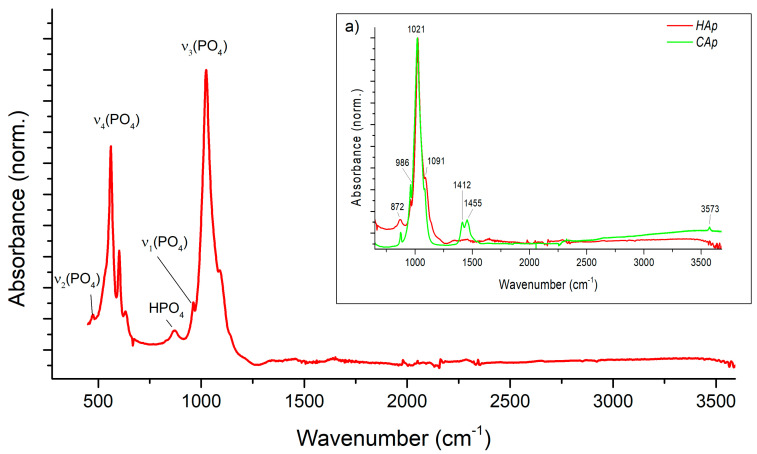
ATR-FTIR spectrum of a typical Hap: (**a**) assessment of precipitated HAp (experiment 5, *f_US_* = 40 kHz; *ε_US_* = 16.46 W/kg; *ε_mix_* = 0.1 W/kg) with commercial HAp (CAp).

**Figure 17:**
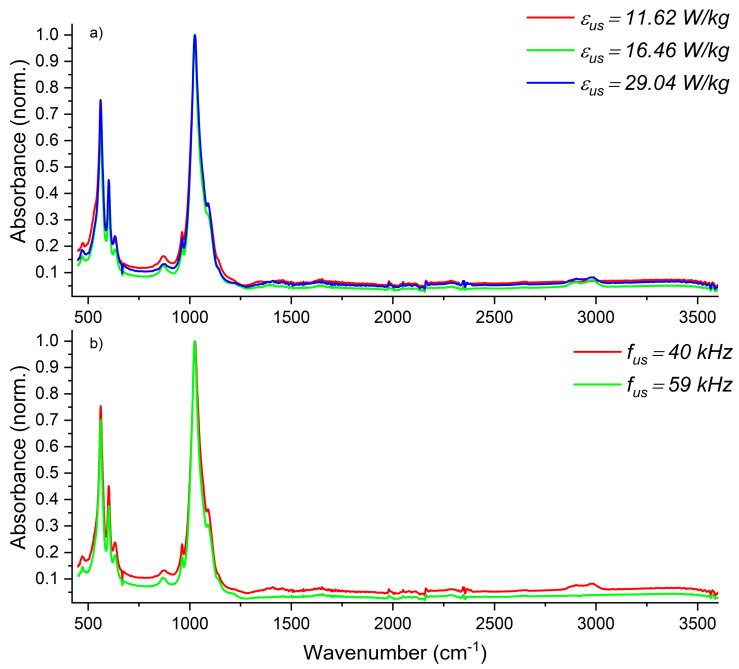
Analysis of the impact of ultrasonic parameters on the ATR-FTIR spectrum and product structure: (**a**) impact of ultrasonic power *ε_US_* (operating conditions: *ε_mix_* = 0.1 W/kg; *f_US_* = 40 kHz); (**b**) impact of ultrasonic frequency *f_US_* (operating conditions: *ε_mix_* = 0.1 W/kg; *ε_US_* = 16.46 W/kg).

**Figure 18:**
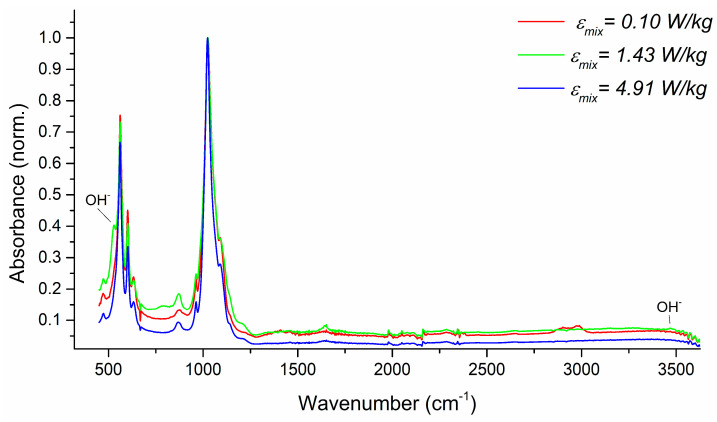
Evaluation of the impact of the unit mixing power *ε_mix_* on the ATR-FTIR spectrum and product structure. Operating conditions: *f_US_* = 40 kHz; *ε_US_* = 16.46 W/kg.

The FT-IR spectra were used to determine the functional groups in the precipitated HAp particles and to check if the obtained product is free from the residual mother liquid. The performed analyses also aimed at the recognition of a type of HAp formed, namely, stoichiometric HAp or its carbonated version CAp (type A with CO_3_^2−^ substituting OH^−^ at 1546 cm^−1^ or type B with CO_3_^2−^ substituting PO_4_^3−^ at 1465 cm^−1^) [39]. The qualitative analysis of the product (Figure 16, Figure 17 and Figure 18) in terms of the impact of both US parameters and mixing conditions has also been performed.

Figure 16 shows a typical ATR-FTIR spectrum of the obtained HAp. The spectrum contains characteristic bands that indicate that the produced HAp is stoichiometric. The main band at 1000–1100 cm^−1^ (Figure 16) corresponds to the asymmetric stretching mode of PO_4_^3−^ groups (υ_3_PO_4_). The shoulder at ~965 cm^−1^ is prescribed to the symmetric stretching (υ_1_PO_4_), while less intense bands at ∼608 and 565 cm^−1^ are due to the bending mode of PO_4_^3−^ groups (υ_4_PO_4_). The band at ∼528 cm^−1^ in the υ_4_PO_4_ domain can be assigned to non-apatitic (surface) HPO_4_^2−^ ions, also clearly visible in Figure 16, which points to the biomimetic nature of the precipitated nanocrystalline apatite. Figure 16a shows a comparison of the obtained product with a commercial HAp (Sigma Aldrich, St. Louis, MO, USA) sample (CAp). In the CAp sample spectrum, there is a narrow peak at ∼3550 cm^−1^, which is characteristic of the hydroxyl group in CAp. The peaks at 1412–1490 cm^−1^ correspond to stretching vibrations in the CO_3_^2−^ group, which confirms that CAp is a carbonated apatite. 

The data presented in Figure 17 show that neither the US power nor the US frequency have any effect on the chemical structure of the obtained HAp.

Figure 18 shows the influence of the unit mixing power, revealing that in the case of the product obtained for *ε_mix_* = 4.91 W/kg, two characteristic OH^−^ bands have appeared at about 3500 cm^−1^ and 630 cm^−1^, which is a characteristic of high crystalline hydroxyapatite [40].

## 5. Conclusions

The presented study took into account the ultrasound-assisted precipitation of hydroxyapatite (HAp) with a view to determining of the effect of operating conditions (together with US parameters) on particles’ characteristics and nucleation kinetics.

Previous observations made by the authors during former CaF_2_ sonoprecipitation studies confirmed the huge influence of mutual relation between mixing conditions and US parameters—most of all ultrasonic power, which affects the number of cavitation bubbles and thus the strength of implosion. The new research, however, also revealed the important impact of particle shape and the ratio between the size of the cavity bubbles and the particles. In the case of needle-shaped, nanosized particles (like HAp), where the linear size of a cavity bubble was about 10^3^ greater than the linear size of a single particle, intense breakage, leading to particles’ fragmentation and significantly increasing the nucleation rate, is affected mainly by mechanical collisions resulting from the increased unit mixing power *ε_mix_*. In turn, for more compact particles, e.g., cubic particles as in the case of CaF_2_, where the size of the cavities and particles is of the same order (micrometers), implosion may be as harmful to particles as mechanical destruction (or even more harmful). In such a case, the destruction comes down to attrition rather than the breakage, and this is why only a limited change in the particles’ shape is observed (in the case of CaF_2_ precipitated in Koflo STM, the particles resembled a spherical shape, as the cube corners were damaged by abrasion).

The optimal conditions of the sonoprecipitation process, oriented to a high-quality product in the form of needles/whiskers/rods (*AR* = 1.8–4), free from agglomerates and with a limited share of particles’ destruction, have been determined. The US parameters, namely the unit power input *ε_US_* = 16.46 W/kg) and the frequency *f_US_* = 40 kHz) have been selected. In turn, mixing conditions *ε_mix_* were determined on the basis of the required particle size. Particles of a mean size *d*~150 nm, in the form of needles/whiskers (*AR*~3.1), a shape similar to HAp in human bones, free from agglomerates, and not fragmented to a great extent (which helps to prevent the risk of secondary sonoagglomeration), could be obtained for *ε_mix_* = 0.1 W/kg. However, if there is a need to use the produced HAp for bone regeneration applications, the mean particle size has to be reduced to the level of ca. 100 nm, which was found to be the value enhancing osteoblast proliferation [12]. Such a result, with a simultaneous narrowing of the *PSD*, may be obtained when *ε_mix_* = 1.47 W/kg. Nevertheless, due to the clogging phenomenon caused mainly by a serious increase in the nucleation rate (about 40 times greater than the nucleation rate in the system without US assistance (Figure 12)), one should include in the reaction system two parallel STMs, working interchangeably and allowing for the reactor cleaning without breaks in continuous work.

A significant intensification of the nucleation stage may be achieved, first of all, through the selection of ultrasonic frequency from a low-frequency region (20–100 kHz). One should be aware that the lower the frequency, the greater the size of the cavities, and the more violent the implosion, which, in turn, means more intense destruction and fragmentation and, ultimately, a greater rate of secondary nucleation. Another important thing is the mutual relation between the ultrasonic power and mixing conditions affecting the turbulence intensity in the reaction system and thus affecting the nucleation stage. The value of the total unit power input (*ε_tot_*) cannot be too high because it may lead to a contrary secondary sonoagglomeration phenomenon. For the examined range of operating conditions, a safe value of *ε_tot_* up to 17 has been determined as a limit by which one may increase the nucleation rate.

Moreover, no negative effects of US assistance on HAp solid purity have been observed. This makes the presented method very competitive, and it is of great importance, especially when compared with methods in which modifiers are used. The additional substances (most of all, different surfactants) used to separate single particles contaminate the main product and are hard to remove. Some of them are even toxic, which excludes their use in contact with living organisms.

Summing up, the sonoprecipitation of HAp particles seems to be an interesting alternative to other commonly used production methods due to its simplicity, low production costs, and high product quality. However, it should be remembered that attentive selection of operating conditions (most of all, US parameters and the unit mixing power) is the key to success, as it may induce one to achieve their goal or, quite the opposite, to miss all of the predictions and assumptions made.

## 6. Patents

The patent describing the method of HAp precipitation in the presence of ultrasounds is pending (patent application P.446658).

## Figures and Tables

**Table 1:** Reactants characteristics.

Parameter	Unit	Starting Solution	
		0.3 M (NH_4_)_2_HPO_4_	0.5 M Ca(NO_3_)_2_
Density *ρ* (t = 20 °C)	kg/m^3^	1023.14	1058.0
Dynamic viscosity *η* (t = 20 °C)	Pa·s	1.102 × 10^−3^	1.071 × 10^−3^
pH (t = 20 °C)	-	initial: 8.3 after correction: 9.5	5.3

**Table 2:** The range of the examined operating conditions.

Lp.	$\dot{V}$ [L/h]	*t_m_* = *τ_US_* * [s]	*ε_mix_* [W/kg]	*f_US_* [kHz]	*P_US_* [W]	*ε_US_* [W/kg]	*ε_tot_* [W/kg]	*Re_eqv_*
1	40	1.61	0.10	40	120	11.62	11.72	5777
2					150	14.52	14.62	6220
3					225	21.78	21.88	7114
4					300	29.04	29.14	7828
5					170	16.46	16.56	6483
6				59			16.56	6483
7	75	0.88	0.63	40			17.09	6552
8	100	0.65	1.47				17.93	6657
9	150	0.43	4.91				21.37	7058

* Values corresponding with the selected fluid dynamic conditions represented by *Re_eqv_.*

## Data Availability

The data is available from the corresponding author upon reasonable request as the patent application is in recognition (current status: pending).

## Nomenclature

$B_{0}=n_{0}\left(\frac{d}{t_{m}}\right)$	nucleation rate [m^−3^s^−1^]
$B_{0_{\mathrm{rel}}}=\frac{B_{0_{\mathrm{mix}}}+B_{0_{\mathrm{US}}}}{B_{0_{\mathrm{mix}}}}$	relative nucleation rate [-]
$Re_{\mathrm{eqv}}={\varepsilon_{tot}}^{\frac{1}{3}}\cdot {d_{\mathrm{eqv}}}^{\frac{4}{3}}\cdot \frac{\rho }{\eta \prime }$	equivalent Reynolds number
$d_{{\mathrm{eqv}}_{\mathrm{STM}}}=\sqrt{\frac{4\cdot V_{\mathrm{STM}}}{\pi \cdot L_{m}}}$	equivalent diameter of a static mixer [m]
$\varepsilon_{\mathrm{tot}}=\varepsilon_{\mathrm{mix}}+\varepsilon_{\mathrm{US}}$	total unit power input [W/kg]
$\varepsilon_{\mathrm{US}}=\frac{P_{\mathrm{US}}}{V_{\mathrm{UB}}\cdot \rho }$	unit power input resulting from the ultrasounds’ use [W/kg]
$\varepsilon_{\mathrm{STM}\;\mathrm{mix}}=\frac{\dot{V}\cdot \Delta p}{V_{\mathrm{STM}}\cdot \rho }$	unit power input resulting from mixing in the static mixer [W/kg]
*B*_0*mix*_	nucleation rate during experiments in silent conditions [m^−3^s^−1^]
*B*_0*US*_	nucleation rate during experiments with US-assistance [m^−3^s^−1^]
*d*	mean particle size [m]
*d_m_*	internal mixer diameter [m]
*F_av_*	average area of particles’ surface [nm^2^]
*f_US_*	ultrasounds frequency [kHz]
*L_m_*	device length [m]
*L*_1_	particle’s length [m]
*L*_2_	particle’s width [m]
*n_b_*	number of cavity bubbles
*n*_0_	nuclei number density per unit volume [1/m^4^]
*P_US_*	power of ultrasounds [W]
Δ*p*	pressure drop [Pa]
*R_b_*	resonant size of cavity bubble [μm]
*t_m_*	residence time in STM [s]
*V_STM_*	volume of STM (excluding static inserts) [m^3^]
*V_UB_*	volume of the ultrasonic bath [m^3^]
$\dot{V}$	volumetric flow rate [l/h]
Greek symbols	
*ε_mix_*	unit power input resulting from mixing [W/kg]
*ε_tot_*	total unit power input [W/kg]
*ε_US_*	unit power input resulting from the ultrasounds’ use [W/kg]
*η*	fluid dynamic viscosity [Pa·s]
*ρ*	fluid density [kg/m^3^]
*τ_US_*	sonication time [s]
Acronyms	
*AR*	aspect ratio
*DTGS*	deuterated triglycine sulfate detector
*FT-IR*	Fourier-transform infrared spectroscopy
*PSD*	particle size distribution
*SEM*	scanning electron microscopy
*STM*	static mixer
*US*	ultrasounds
*XRD*	X-ray diffraction
